# Evaluation of the Wound Healing Potential of *Achillea biebersteinii* Afan. (Asteraceae) by *In Vivo* Excision and Incision Models

**DOI:** 10.1093/ecam/nep039

**Published:** 2011-06-16

**Authors:** Esra Küpeli Akkol, Ufuk Koca, Ipek Pesin, Demet Yilmazer

**Affiliations:** ^1^Department of Pharmacognosy, Faculty of Pharmacy, Gazi University, Etiler 06330, Turkey; ^2^Department of Pathology, Dışkapı Yıldırım Beyazit Education and Research Hospital, Ankara, Turkey

## Abstract

*Achillea* species are widely used for diarrhea, abdominal pain, stomachache and healing of wounds in folk medicine. To evaluate the wound healing activity of the plant, extracts were prepared with different solvents; hexane, chloroform, ethyl acetate and methanol, respectively from the roots of *Achillea biebersteinii*. Linear incision by using tensiometer and circular excision wound models were employed on mice and rats. The wound healing effect was comparatively evaluated with the standard skin ointment Madecassol. The *n*-hexane extract treated groups of animals showed 84.2% contraction, which was close to contraction value of the reference drug Madecassol (100%). On the other hand the same extract on incision wound model demonstrated a significant increase (40.1%) in wound tensile strength as compared to other groups. The results of histoptological examination supported the outcome of linear incision and circular excision wound models as well. The experimental data demonstrated that *A. biebersteinii* displayed remarkable wound healing activity.

## 1. Introduction

The genus *Achillea* (Asteraceae), named after the mythological Greek warrior Achilles, who used *Achillea* species for healing wounded-soldiers during the Trojan War [1]. The genus *Achillea* comprises of *∼*85 species, most of which are endemic to Europe and the Middle East. Turkish flora possesses 42 *Achillea* species and 23 of them are endemic [2]. These species have some interesting properties and are used in cosmetics, fragrances and agriculture, for example, plant protection [3]. Some *Achillea* species have been known to be ethnopharmacologically used in folk remedies for various purposes such as hemorrhoid and wound healing [4]. Herbal teas prepared from some *Achillea* species are very often used in folk medicine as diuretic, for abdominal pain, against diarrhea, flatulence and emmenagog, moreover for wound healing purposses, in Turkey [5–7].

*Achillea biebersteinii* is locally named Sarıçiçek in Turkish, and other species widely used as a folk remedy to treat abdominal pain, wounds and stomachache as well [8, 9]. *A. biebersteinii* Afan. [Asteraceae, Section: Filipendulinae (D.C.) Boiss] (syn. *A. micrantha*) is a perennial herb, villose, stems erect, simple or branched from the base; 30–60 cm high; leaves up to 10 cm, oblong-lanceolate in outline, pinnatisect into numerous narrow segments, segments divided into minute linear-lanceolate mucronate lobes; the heads are radiate, in large dense compound corymbs; involucre 4-5 mm, oblong-ovoid; flowering period, April-May.

Several biological activity studies have been performed on various *Achillea* species, including antibacterial, antioxidant, anti-inflammatory and antispasmodic activities [10–13].

The aim of the present study was to investigate the *in vivo* wound healing activity of *A. biebersteinii* in order to elucidate traditional use of this plant from the scientific point of view. The hexane, chloroform, ethyl acetate and methanolic extracts prepared from the aerial parts of the mentioned plant were tested in mice and rats for wound healing activity using *in vivo* circular excision and linear incision wound models.

## 2. Materials and Methods

### 2.1. Plant Material

*Achillea biebersteinii* Afan. aerial parts were collected from Beynam forest, Ankara, Turkey, in May 2007 and was identified by Prof. Dr M. Vural from the Department of Botany, Faculty of Art and Science, Gazi University. A voucher specimen was deposited in the Herbarium of Faculty of Pharmacy, Gazi University (GUE-2602).

### 2.2. Extraction

Following dissection of the aerial parts of *A. biebersteinii* Afan., they were shade dried. Each 30 g aerial parts was submitted to successive solvent extractions seperatelly with *n*-hexane, chloroform, ethyl acetate and methanol at room temperature for a day (500 ml each solvent). This was repeated in two trials. After filtration, the extracts were evaporated at 40°C (Buchi, Switzerland) to dryness *in vacuo*. Yields of each extracts were 7.9% for *n*-hexane, 4.8% for diethyl ether, 1.3% for ethyl acetate and 15.3% for methanol.

### 2.3. GC-MS Analysis of the *n*-Hexane Fraction

Chromatographic analysis was carried out on Agilent 6890N Network GC system combined with Agilent 5973 Network Mass Selective Detector (GC-MS). The capillary column used was an HP 5 MS Column (30 m × 0.25 mm × 0.25 *μ*m). Helium was used as carrier gas at a flow rate of 1 ml/min with 1 *μ*l injection volume. Samples were analyzed with the column held initially 60°C for 1 min after injection with 1 min hold time, then increased to 180°C with 3°C/min heating ramp and kept at 180°C for 20 min. The injection was performed in splitless mode. Detector and injector temperatures were 280°C and 250°C, respectively. Run time was 60 min. MS scan range was (*m/z*): 35–500 atomic mass units (AMU) under electron impact (EI) ionization (70 eV).

### 2.4. Animals

Male, Sprague-Dawley rats (160–180 g body weight) and Swiss albino mice (20–25 g body weight) were purchased from the animal breeding laboratories of Refik Saydam Central Institute of Health (Ankara, Turkey).

The animals left for 3 days at room conditions for acclimatization. They were maintained on standard pellet diet and water *ad libitum* throughout the experiment. A minimum of six animals were used in each group, otherwise described in procedure. The study was permitted by the Institutional Animal Ethics Committee (Gazi University Ethical Council Project Number: G.U.ET-08.037) and was performed according to the international rules considering the animal experiments and biodiversity right.

### 2.5. Wound Healing Activity

Incision and excision wound models were used to evaluate the wound healing activity. For the *in vivo* wound models, samples were prepared in the ointment consists of propylene glycol : liquid paraffin (6 : 1) and applied topically onto the test animals Extracts were prepared as 1% in the ointment, and 0.5 mg of it applied on the wound sides immediately after wound was created artificially.

The vehicle group of animals was treated with the ointment only. The negative control group of animals was not treated with a material. Commercial Madecassol, 0.5 g (Bayer) was applied as a reference drug.

### 2.6. Linear Incision Wound Model

All the animals were anaesthetized with 0.15 cc Ketalar and the back hair of the rats were shaved by using a shaving machine. Five centimeters long, two linear-paravertebral incisions were made with a sterile blade through the full thickness of the skin at the distance of 1.5 cm from the midline of each side of the vertebral column [14]. The wounds were closed with three surgical interrupted sutures of 1 cm apart. The animals were divided into four groups. The extracts, the reference material (Madecassol) and the vehicle were topically applied once in a day through 9 days. The fourth group, negative control group of animals, was not treated with a material. All the sutures were removed on the 9th post wound day. On day 10 all the animals were killed with ether anesthesia. One linear-paravertebral incised skin was measured using tensiometer (Zwick/Roell Z0.5, Germany) for its tensile strength, the other incised skin was sent for histopathological examination [15, 16].

### 2.7. Excision Wound Model

This model was used to monitor wound contraction and wound closure time. Each group of animals (six-animal each) was anaesthetized by 0.01 cc Ketalar. The back hairs of the mice were depilated by shaving. The circular wound was created on the dorsal interscapular region of each animal by excising the skin with a 5 mm biopsy punch; wounds were left open [17]. The extracts, the reference material (Madecassol Bayer) and the vehicle ointment were administered once a day till the wound was completely healed. The progressive changes in wound area were monitored by a camera (Fuji, S20 Pro, Japan) every other day. Later on, wound area evaluated by using AutoCAD program. Wound contraction was calculated as percentage of the reduction in wounded area. A specimen sample of tissue was isolated from the healed skin of each group of mice for the histopathological examination [18].

### 2.8. Histological Study

Sample tissues were fixed in 10% formalin and were embedded in paraffin wax. Serial sections (5 *μ*m thickness) of paraffin embedded tissues were cut. The tissues were stained by haematoxylin and eosin, which were examined by light microscope (Olympus BX51). Ulceration, necrosis and epithelisation were evaluated in the skin tissues. Also congestion, edema, PNL, mononuclear cells, fibroblasts and vascularisation were qualitatively evaluated as −, +, ++ and +++.

### 2.9. Statistical Analysis of the Data

The data on percentage wound healing was statistically analyzed using one-way analysis of variance (ANOVA). The values of *P* ≤  .001 were considered statistically significant.

Mann-Whitney *U*, Kruskal-Wallis and chi-squared tests were used for the statistical analysis of the histopathological data.

## 3. Results

### 3.1. Excision and Incision Wound Models

In this study, an enquiry on wound healing activity of the *n*-hexane, chloroform, ethyl acetate and methanol extracts prepared from aerial parts of *A. biebersteinii*, which has been used in the treatment of wounds was carried out on mice and on rats by excision and linear incision wound models to verify the claimed traditional use of the plant on a scientific base. More to the point, histopathologic examination of the same extracts were also assessed.

**Figure 1:**
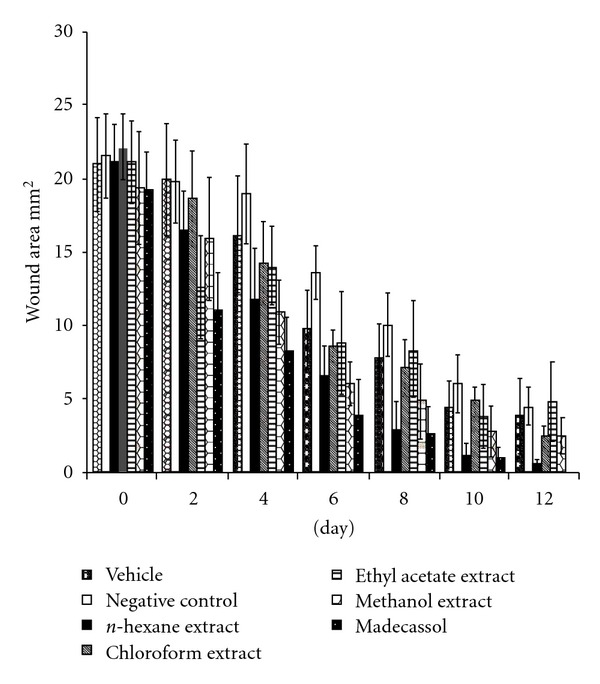
Effects of the extracts from *A. biebersteinii* on circular excision wound model.

The measurements of the progress of wound healing induced by the extracts, reference drug, negative control and vehicle groups in the excision wound model are shown in Figure 1. The *n*-hexane extract treated groups of animals showed 61.7% (*P* <  .01) contraction on the wounds on day eight. This extract demonstrated 84.2% (*P* <  .001) contraction on day twelfth, which was close to contraction value of the reference drug Madecassol^®^ (100%). On the given days, other extracts showed no significant results.

**Figure 2:**
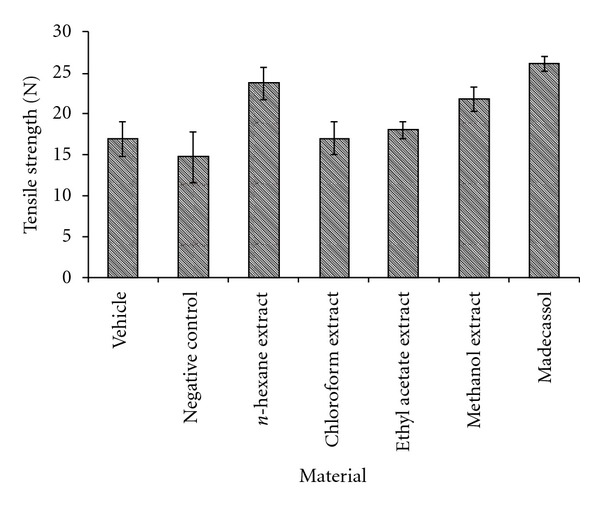
Effects of the extracts from *A. biebersteinii* on linear incision wound model.

The results of the measurements of tensile strength are shown in Figure 2. Tensile strength of the animals treated with *n*-hexane extract demonstrated the highest value (40.1%, *P* <  .001) on day 10. Topical application of the *n*-hexane extract on incision wound model demonstrated a significant increase in wound tensile strength as compared to other groups.

### 3.2. Histological Study

**Figure 3:**
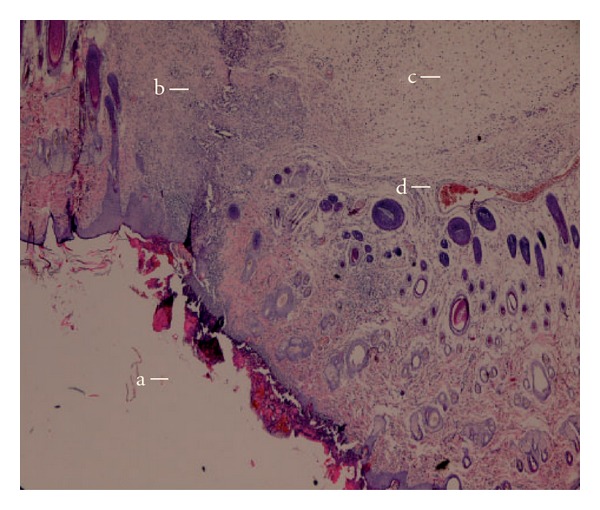
Microscopic view of the section of negative control group (untreated) 10 days old wound tissue. (a) area of ulceration; (b) mixed type inflammatory cells; (c) edema and (d) congested vessel.

**Figure 4:**
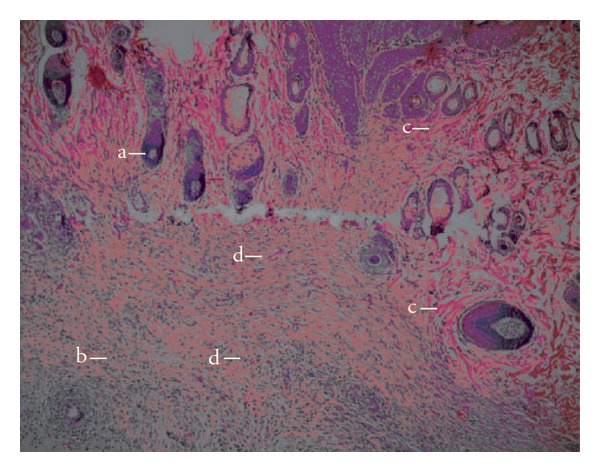
Microscopic view of the section of 10 days old wound tissue treated with *n*-hexane extract. (a) hair follicle; (b) fibroblast; (c) collagen fibres and (d) blood vessel.

**Figure 5:**
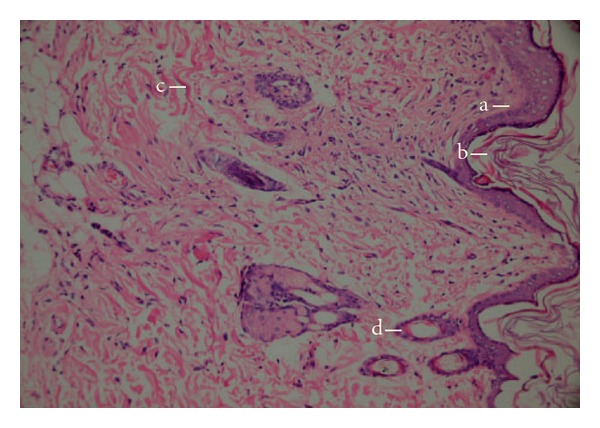
Microscopic view of the section of 10 days old wound tissue treated with Reference material Madecassol^®^ (a) intact epidermis; (b) keratinization; (c) collagen fibers and (d) hair follicle.

**Figure 6:**
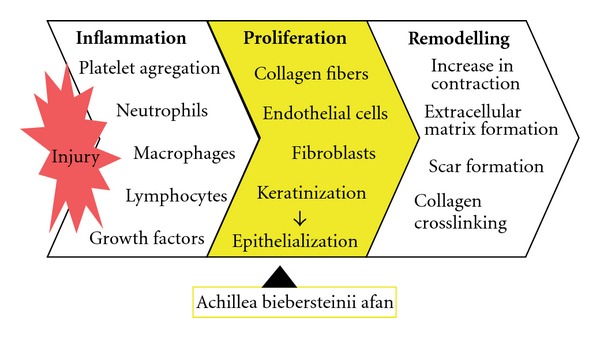
The hypothetical diagram demonstrating the possible effect of the extract of *A. biebersteinii* in wound healing activity.

Histopatological examinations of the negative control exhibited wide area of ulceration containing fibrinous exudate and inflammatory cells, mild degree inflammation and mixed type inflammatory cells in dermis, slightly increased vascularization with congestion, which alltogether show that the healing was not completed on the wounded area (Figure 3). When the *n*-hexane extract treated group was histopathologically analyzed, establishment of collagen fibers, fibroblasts and mature hair follicles were obviously seen on the representing picture (Figure 4). Moreover, no scar tissue on the dermis in *n*-hexane treated group exhibits that the healing was almost completed. In a similar way, group treated with Madecassol showed mature epidermis with keratinization and mature hair follicles, fibroblasts in dermis that are the proof of completion of healing (Figure 5). Histological observation suggested that the phytochemical content of the n-hexan extract might be responsible for collagen formation at the proliferative state, which is contributed by increased fibroblats content (Figure 6).

## 4. Discussion

Overall the histopathological examinations showed that healing process of the wounded tissue in n-hexane treated group was comparably close to the reference drug treated group, whereas no healing was observed in negative control group (untreated). Granulation tissue primarily contains fibroblasts, collagen fibres, very less edema and newly generated blood vessels, which were also observed in *n*-hexane extract treated group of animals. This histopathological observation provided additional evidence for the experimental wound healing studies based on the contraction value of wound areas and the measurement of tensile strength (Figures 3–5).

In the present study, the *n*-hexane extract of *A. biebersteinii* aerial parts was found to be remarkably active on *in vivo* wound models, whereas the other extracts were not as active as the *n*-hexane extract. This prompted us to analyze and identify chemical composition of the *n*-hexane extract directly by capillary GC-MS. Analysis of the *n*-hexane fraction revealed that the major compounds were determined as sesquiterpenoid *β*-Eudesmol, piperitone, camphor, borneol, *α*-terpinene, 1,8-cineole, although their amounts were 19.1%, 9.2%, 4.2%, 3.3%, 2.9% and 2.5% respectively representing 100% of the extract (Table 1).

*Achillea* species have been so far reported to contain diterpenes, sesquiterpenes, flavonoids, lignans, essential oil and rarely triterpenes [19–26]. For instance, *A. vermicularis* was shown to have guaianolide- and germacrene-type sesquiterpenes as well as flavonoids, whereas *A. setacea* was reported to contain sesquiterpenes, essential oils and flavonoids [20, 21, 27]. In addition to extracts, essential oils of the *Achillea* species were also analysed. The oil of *A. pachycephala* was found to contain 1,8-cineole and camphor as the major constituents, whereas 1,8-cineole and artemisia ketone were major in *A. oxyodonta*. On the other hand *A. biebersteinii* was rich in camphor and borneol followed by 1,8-cineole. It was stated that all the oils were rich in oxygenated monoterpenes [28]. Non-volatile components of *A. biebersteinii* afforded in addition to *β*-sitosterol, stigmasterol two sesquiterpene lactones, germacranolide [29]. Essential oil of *A. millefolium* consists of a number of monoterpenes such as *α*-pinene, *β*-pinene, 1,8-cineole, camphor and borneol in addition to some sesquiterpene lactones of germacrene-derivatives [25]. Major component in the essential oils of both *A. setacea* and *A. teretifolia* was elucidated to be 1,8-cineole [27] whereas *α*-pinene, 1,8-cineole and camphor as well as germacrene D and bisabolene as the major constituents of ten other *Achillea* species (*A. biserrata*, *A. clypeotala*, *A. crithmifolia*, *A. filipendula*, *A. macrophylla*, *A. pannonica*, *A. pyrenaica*, *A. sibirica*, *A. taygetea* and *A. tenuifolia*) [23].

Various biological activity studies were also completed on *Achillea* species. The antimicrobial and antioxidant activities of the essential oil and the methanolic extract of *A. biebersteinii* were studied *in vitro* by Baris et al. [30]. The essential oil showed antimicrobial activity against 8 bacteria sp., 14 fungi sp. and the *C. albicans*, whereas the methanolic extract remained inactive. GC-MS analysis of the essential oil resulted in the identification of 64 components representing 92.24% of the oil, piperitone, camphor and 1,8-cineole (eucalyptol) as being major constituents [30]. In another study, antimicrobial activity tests carried out with the fractions of the oil revealed that the major activity was observed in those contain 1,8-cineole and camphor, followed by borneol and piperitone [31].

When a wound occurs and is exposed to external environment, it is more prone to attack by microbes, which invade through the skin and delay the natural wound healing process. Reactive oxygen species (ROS), are vital part of healing and serve as cellular messengers that drive numerous aspects of molecular and cell biology. ROS can trigger the various beneficial pathways of wound healing, for example, at micromolar concentrations of hydrogen peroxide can promote vascular endothelial growth factor (VEGF) expression in keratinocytes [32, 33]. During the inflammation phase of healing neutrophils and macrophages are attracted into the injured tissue by various chemotactic factors. They locate, identify, phagocytize, kill and digest microorganisms and eliminate wound debris through their characteristic “respiratory burst” activity and phagocytosis [34]. At high concentrations, ROS can induce severe tissue damage and even lead to neoplastic transformation, which further impede the healing process by causing damage to cellular membranes, DNA, proteins and lipids as well [35]. Hence, if a compound or a plant extract having antioxidant potentials and antimicrobial activity additionally, it can be a good therapeutic agent for accelerating the wound-healing process.

Several preparations containing *A. millefolium* extract was quite successfully healed the wounds and scars. The liniment containing hiperisin oil and *A. millefolium* extract patented by Motogna accelerates the healing of wounds and gives esthetic scars. Since the liniment is applied as a spray it is easily applied and painless [36]. The activiy most probably comes from the synergistic effect of compounds present in the extract and also additive effect of hiperisin.

According to ethnopharmacological studies botanical remedies provide two advantages over single-compound drugs: primary active compounds in plants are synergized by secondary compounds and secondary compounds ease the side effects caused by primary active compounds. The course of searching an ethnopharmacologically active plant extract down to a single active principal may result in a defeat of biological activity for a number of reasons, for instance, a special compound might be unstable during extraction, fractionation or in the purified form, or, the fundamental basis for ethnopharmacology does not always exist in a single active compound but rather is a result of the interaction of more than one active compounds found in the extract [37]. Moreover, that single compound might potentiate the activity and that single compound become toxic compared to whole plant extract [38]. Thus, the likelihood that more than one compound present in a plant extract could contribute to a net pharmacological response of the extract.

## 5. Conclusion

According to results reported here n-hexane extract of *Achillea biebersteinii* was found to have better activity on the wound healing experimental models compared to the other extracts. Even though the analysis of the n-hexane extract revealed that the major compounds were determined as sesquiterpenoid *β*-Eudesmol, piperitone, additional compounds, such as camphor, borneol, *α*-terpinene and 1,8-cineole were also determined. This unveils that the extract was not dominated by a single compound, whereas the extract contains various effective compounds in different quantities. This may suggest that *β*-Eudesmol and piperitone solely could not be reason for the activity but mostly synergistic interaction with the rest of the ingredients might be promoting the wound healing activity. This study provides scientific evidence to the ethnomedicinal futures of *A. biebersteinii*.

## Figures and Tables

**Table 1:** GC-MS analysis results of *n*-hexane fraction.

Components identified	Retention times (*R*_t_, min)	Composition (%) *n*-hexane fr.
1,8-Cineole	7.762	2.531
Linalool	10.232	0.167
Camphor	11.909	4.244
Borneol	12.783	3.335
Terpinen-4-ol	13.269	0.439
*α*-Terpinene	13.836	2.989
Piperitone	16.487	9.190
Bicyclo [2,2,1]heptane-2,5-dione	16.808	0.305
1,7-Octadiene-3,6-diol	17.370	0.399
Thymol	18.227	0.240
Tridecane	18.482	0.387
4-Hydroxy-2-methylacetophenone	18.622	0.286
2,4-Heptadien-1-ol	20.412	0.262
(E)-İsoeugenol	20.824	0.282
*α*-Amorphene	21.131	0.641
*α*-Copaene	21.531	1.262
Decanoic acid	21.673	0.981
Tetradecane	22.655	0.640
Cadinene	27.108	0.496
2-(4*H*)-Benzofuranone	27.596	0.785
2-Cyclopenten-1one	29.254	0.388
Caryophyllene oxide	29.719	3.123
Hexadecane	30.542	4.220
Aromadendrene	31.482	0.480
*δ*-Selinene	31.592	0.504
Caryophylla-4(14), 8(15)-dien-5-beta-ol	31.737	1.162
*β*-Eudesmol	32.293	19.111
Alloaromadendrene	32.549	0.756
Vulgarol B	33.565	0.506
3-Buten-2-one	33.695	0.297
Cyclodecane	34.011	0.633
(–)-Loliolide	36.380	0.580
*Cis*,*cis*-7,10,-Hexadecadienal	37.195	0.416
Octadecane	37.746	1.110
Oxirane	38.274	0.666
Cyclohexanone	39.057	0.381
Cyclohexadecanolide	39.222	5.544
Benzylsalicylate	39.852	1.137
Phthalic acid	39.982	2.590
Tetradecene	40.402	1.302
1-Methyldodecylbenzene	41.282	0.463
Hexadecanoic acid	43.632	1.338
Cyclohexane	44.606	0.959
1,15-Hexadecadiene	45.874	0.480
Oleic acid	46.797	0.536
*n*-Hexadecanol	49.275	0.991
Heneicosane	50.396	0.887
Phytol	51.125	2.941
Methyl linoleate	52.309	3.028
Others	—	13.61

Total		100
